# Supplementary material for: Synthesis and Antibacterial Activity of Polyalthic Acid Analogs
Source: Antibiotics (Basel). 2023 Jul 19;12(7):1202. doi: 10.3390/antibiotics12071202 (PMC10376133; doi:10.3390/antibiotics12071202)
Supplement: Supplementary file 1 [file antibiotics-12-01202-s001.zip › antibiotics-2467226-supplementary.pdf]

Supporting Information

Synthesis and Antibacterial Activity of Polyalthic Acid Analogs

Table of contents:

Susceptibility profile of the Gram-positive and Gram-negative strains tested in this study using antibiotics currently available in the market.

Determination of MIC of PA, daptomycin, **2a** and **3a** by analysis of the kinetic growth of gram-positive bacteria

^{1}H and ^{13}C NMR data

Table S1. Susceptibility profile of the Gram-positive strains tested in this study using antibiotics currently available in the market.

Antibiotics	*S. epidermidis* ATCC 35984		*S. aureus* ATCC 25923		*S. aureus* ATCC 8095		*E. faecalis* ATCC 292	
	MIC	interpretation	MIC	interpretation	MIC	interpretation	MIC	interpret
Ampicillin	>4	R	N.R.	N.R.	N.R.	N.R.	≤2	S
Cefoxitin	≤0.5	R	≤1	S	2	S	N.R.	N.R.
Ceftaroline	N.R.	N.R.	≤0.5	S	≤0.5	S	N.R.	N.R.
Ciprofloxacin	≤0.5	I	≤0.5	S	≤0.5	S	1	S
Clindamycin	>2	R	≤0.5	S	≤0.5	S	N.R.	N.R.
Chloramphenicol	N.R.	N.R.	8	S	8	S	N.R.	N.R.
Daptomycin	≤1	S	≤1	S	≤1	S	N.R.	N.R.
Erythromycin	>4	R	≤0.25	S	≤0.25	S	N.R.	N.R.
Streptomycin-Sin	N.R.	N.R.	N.R.	N.R.	N.R.	N.R.	>1000	R
Gentamicin	>8	R	≤2	S	≤2	S	N.R.	N.R.
Gentamicin-Sin	N.R.	N.R.	N.R.	N.R.	N.R.	N.R.	≤500	S
Linezolid	2	S	≤1	S	≤1	S	2	S
Minocycline	≤1	S	≤1	S	≤1	S	N.R.	N.R.
Nitrofurantoin	N.R.	N.R.	N.R.	N.R.	N.R.	N.R.	≤16	S
Oxacillin	>2	R	≤0.25	S	0.5	S	N.R.	N.R.
Penicillin G	>1	R	≤0.125	S	≤0.125	S	N.R.	N.R.
Teicoplanin	≤2	S	≤1	S	≤1	S	≤1	S
Trimethoprim-Sulfamethoxazole	>2/38	R	≤0.5/9.5	S	≤0.5/9.5	S	N.R.	N.R.
Vancomycin	1	S	1	S	1	S	2	S

*N.R. – not reported, either because the MIC is reported only with no interpretation, or the test is not reported because there is insufficient information to have a breakpoint; S -Susceptible; I- Susceptible increased exposure; R- Resistant.

Table S2. Susceptibility profile of the Gram-negative strains tested in this study using antibiotics currently available in the market.

*N.R. – not reported, either because the MIC is reported only with no interpretation, or the test

Antibiotics	*K. pneumoniae* ATCC 700603		*E. coli* ATCC 25922		*A. baumannii* ATCC 1960[illegible]	
	MIC	interpretation	MIC	interpretation	MIC	interpretatio[illegible]
Amikacin	≤4	S	≤4	S	>16	R
Ampicillin-Sulbactam	16/8	I	≤4/2	S	N.R.	N.R.
Cefepime	2	S	≤1	S	N.R.	N.R.
Ceftazidime	>8	R	≤1	S	N.R.	N.R.
Ceftazidime-Avibactam	1/4	S	≤0.25/4	S	N.R.	N.R.
Ceftolozano-Tazobactam	≤1/4	S	≤1/4	S	4/4	N.R.
Ceftriaxone	4	R	≤1	S	N.R.	N.R.
Cefuroxime	>16	R	≤4	S	N.R.	N.R.
Ciprofloxacin	0.5	I	≤0.25	S	1	I
Colistin	≤1	S	≤1	S	≤1	S
Ertapenem	≤0.25	S	≤0.25	S	N.R.	N.R.
Gentamicin	>4	R	≤1	S	>4	R
Imipenem	≤0.25	S	≤0.25	S	8	R
Levofloxacin	1	I	≤0.5	S	≤0.5	S
Meropenem	≤0,5	S	≤0.5	S	1	S
Piperacillin-Tazobactam	16/4	S	≤4/4	S	N.R.	N.R.
Trimethoprim-Sulfamethoxazole	≤1/19	S	≤1/19	S	>4/76	R

is not reported because there is insufficient information to have a breakpoint; S -Susceptible; I-Susceptible increased exposure; R- Resistant.

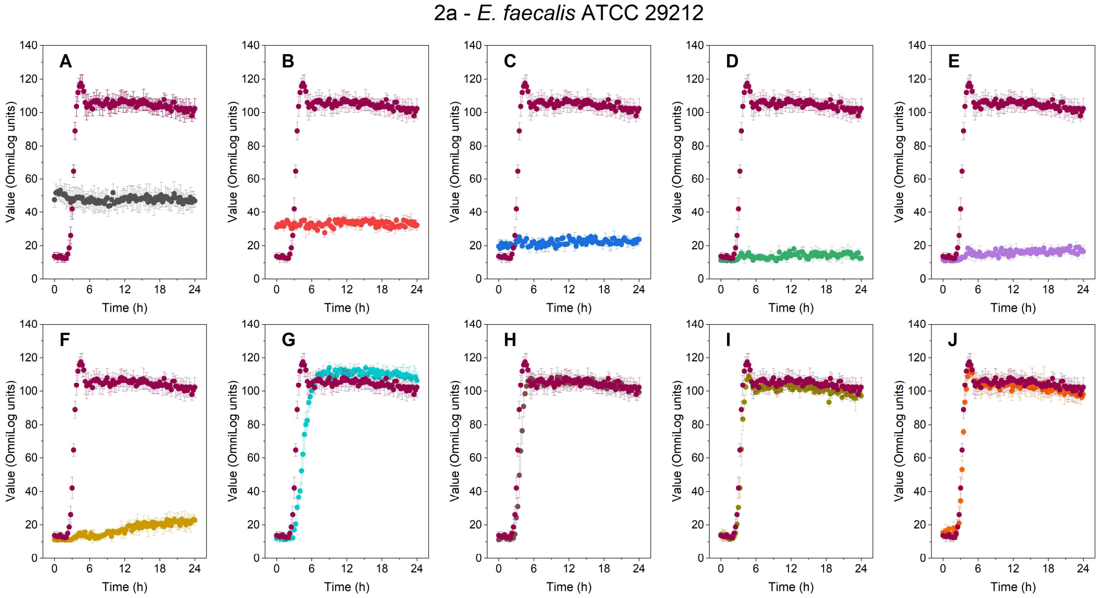

Figure S1. Determination of the MIC of **2a** against *E. faecalis* ATCC 29212 by a kinetic growth assay. A - 512 mg/L, B - 256 mg/L, C - 128 mg/L, D - 64 mg/L, E - 32 mg/L, F - 16 mg/L, G - 8 mg/L, H - 4 mg/L, I - 2 mg/L, J - 1 mg/L. The MIC was observed at 16 mg/L.

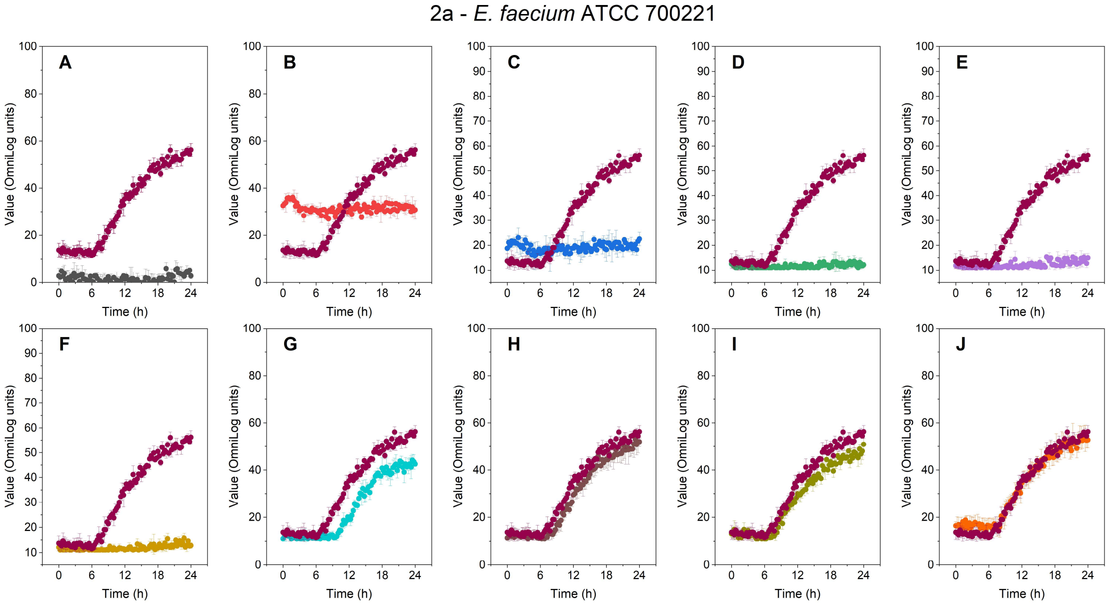

Figure S2. Determination of the MIC of **2a** against *E. faecium* ATCC 700221. A - 512 mg/L, B - 256 mg/L, C - 128 mg/L, D - 64 mg/L, E - 32 mg/L, F - 16 mg/L, G - 8 mg/L, H - 4 mg/L, I - 2 mg/L, J - 1 mg/L. The MIC was observed at 16 mg/L.

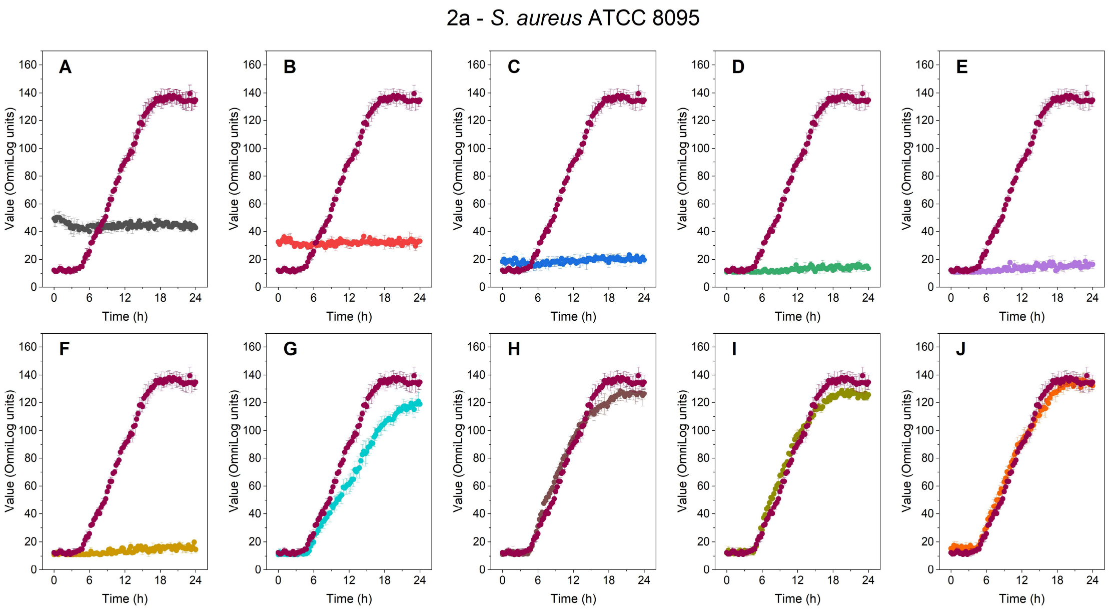

Figure S3. Determination of the MIC of **2a** against *S. aureus* ATCC 8095. A - 512 mg/lL, B - 256 mg/L, C - 128 mg/L, D - 64 mg/L, E - 32 mg/L, F - 16 mg/L, G - 8 mg/L, H - 4 mg/L, I - 2 mg/L, J - 1 mg/L. The MIC was observed at 16 mg/L.

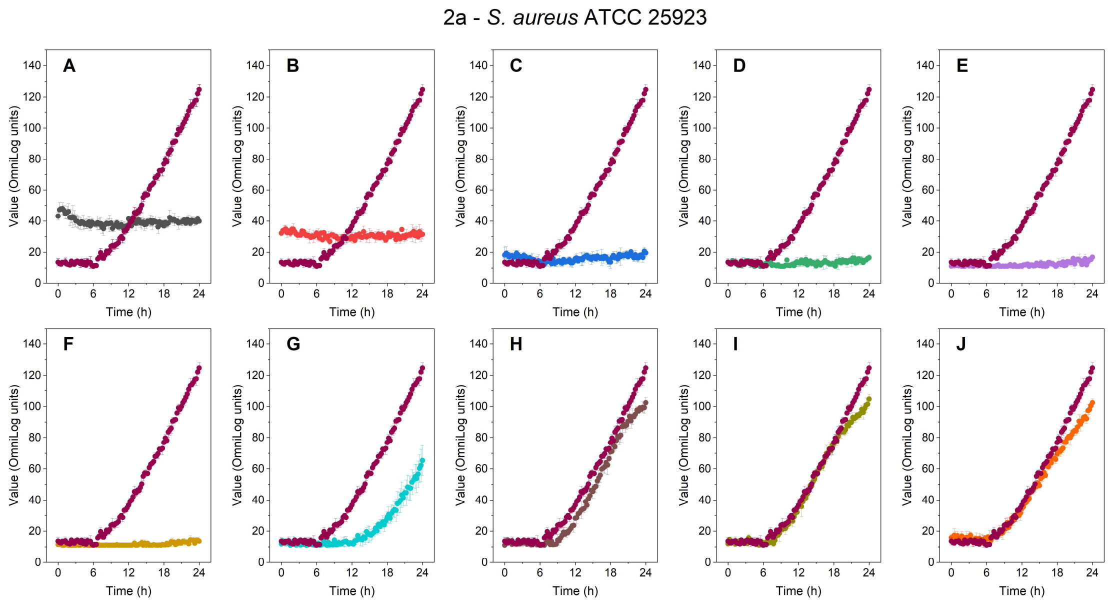

Figure S4. Determination of the MIC of **2a** against *S. aureus* ATCC 25923. A - 512 mg/L, B - 256 mg/L, C - 128 mg/L, D - 64 mg/L, E - 32 mg/L, F - 16 mg/L, G - 8 mg/L, H - 4 mg/L, I - 2 mg/L, J - 1 mg/L. The MIC was observed at 16 mg/L.

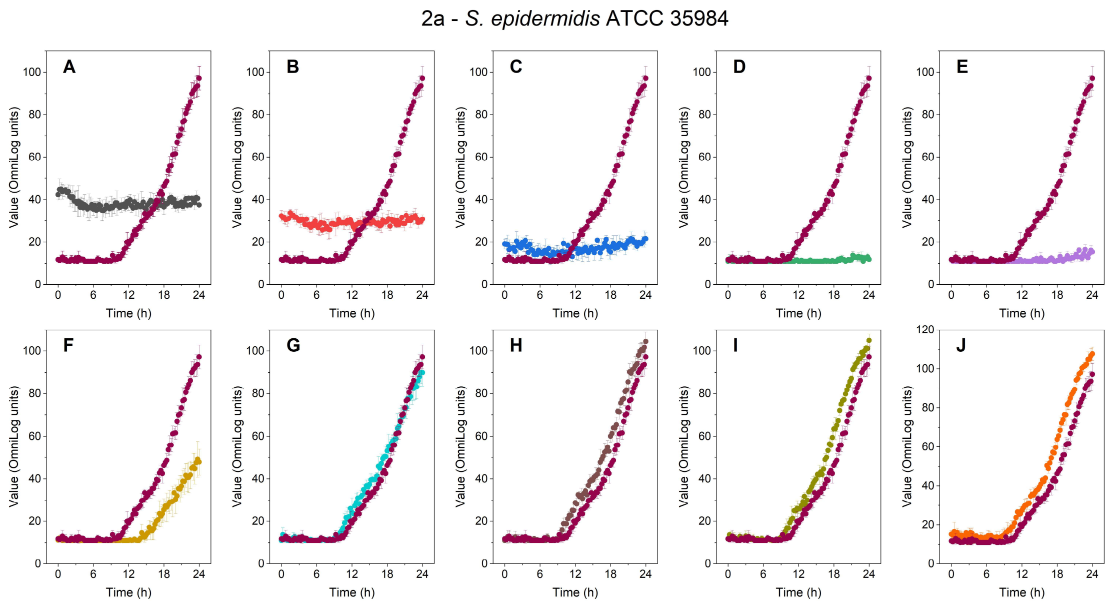

Figure S5. Determination of MIC of **2a** against *S.epidermids* ATCC 35984. A - 512 mg/L, B - 256 mg/L, C - 128 mg/L, D - 64 mg/L, E - 32 mg/L, F - 16 mg/L, G - 8 mg/L, H - 4 mg/L, I - 2 mg/L, J - 1 mg/L. The MIC was observed at 32 mg/L.

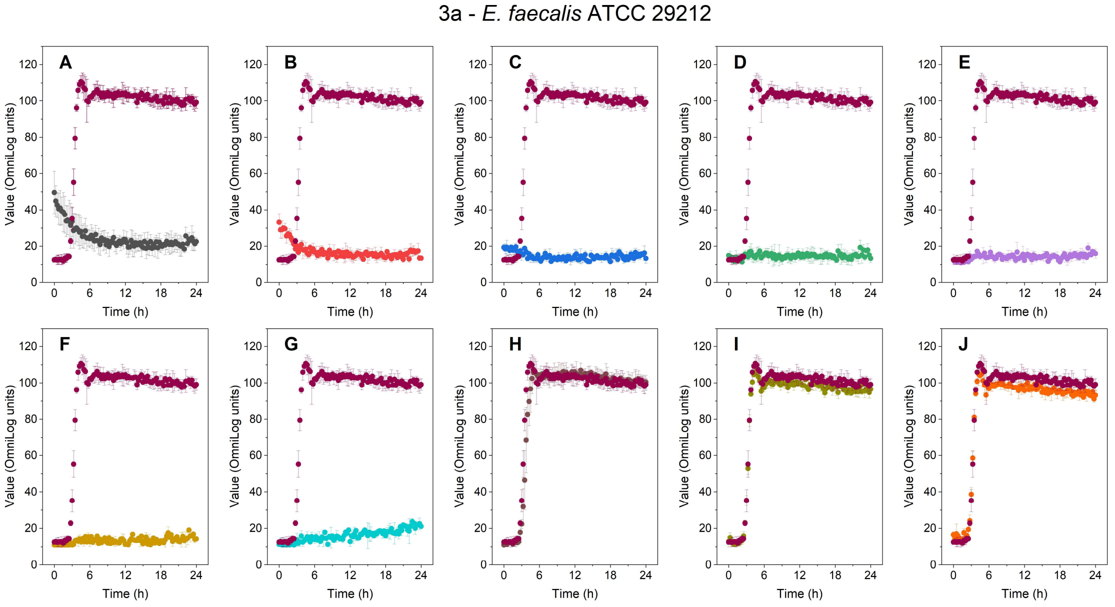

Figure S6. Determination of the MIC of **3a** against *E. faecalis* ATCC 29212. A - 512 mg/L, B - 256 mg/L, C - 128 mg/L, D - 64 mg/L, E - 32 mg/L, F - 16 mg/L, G - 8 mg/L, H - 4 mg/L, I - 2 mg/L, J - 1 mg/L. The MIC was observed at 8 mg/L.

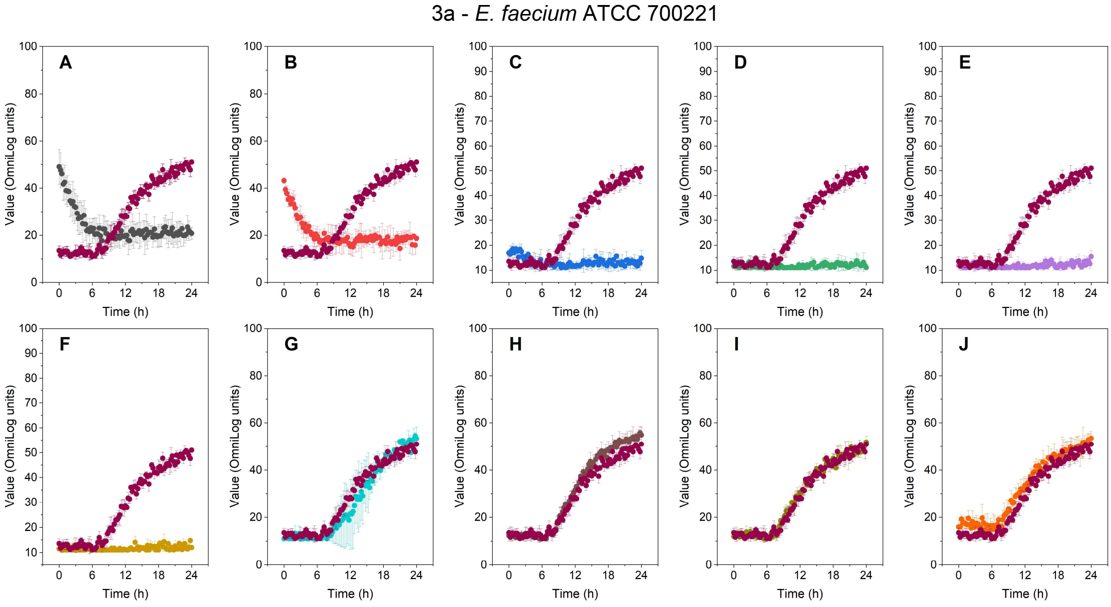

Figure S7. Determination of the MIC of **3a** against *E. faecium* ATCC 700221. A - 512 mg/L, B - 256 mg/L, C - 128 mg/L, D - 64 mg/L, E - 32 mg/L, F - 16 mg/L, G - 8 mg/L, H - 4 mg/L, I - 2 mg/L, J - 1 mg/L. The MIC was observed at 16 mg/L.

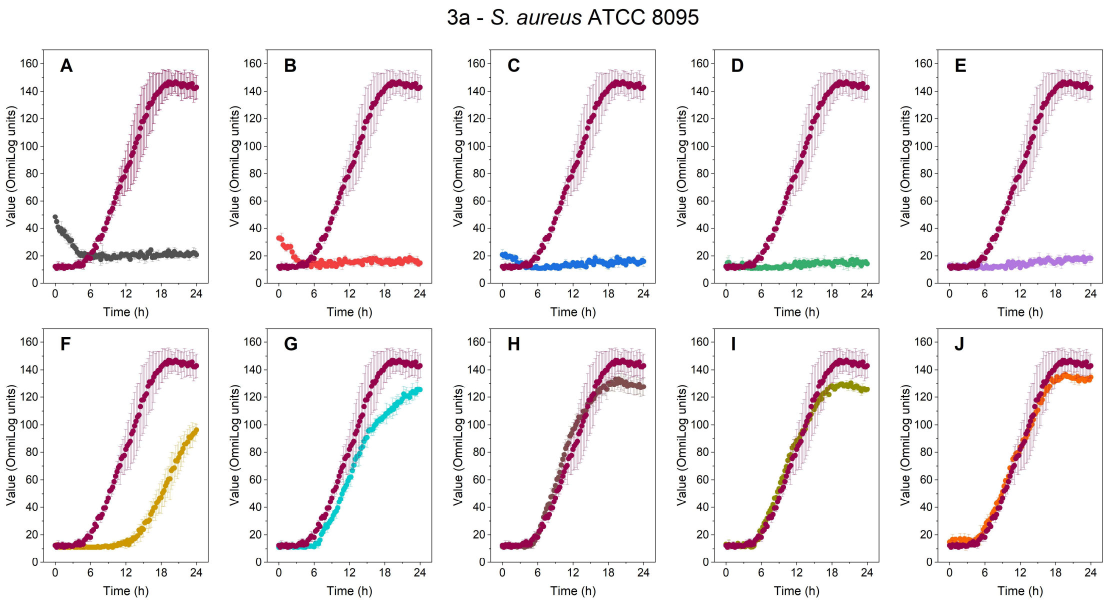

Figure S8. Determination of the MIC of **3a** against *S. aureus* ATCC 8095. A - 512 mg/L, B - 256 mg/L, C - 128 mg/L, D - 64 mg/L, E - 32 mg/L, F - 16 mg/L, G - 8 mg/L, H - 4 mg/L, I - 2 mg/L, J - 1 mg/L. The MIC was observed at 32 mg/L.

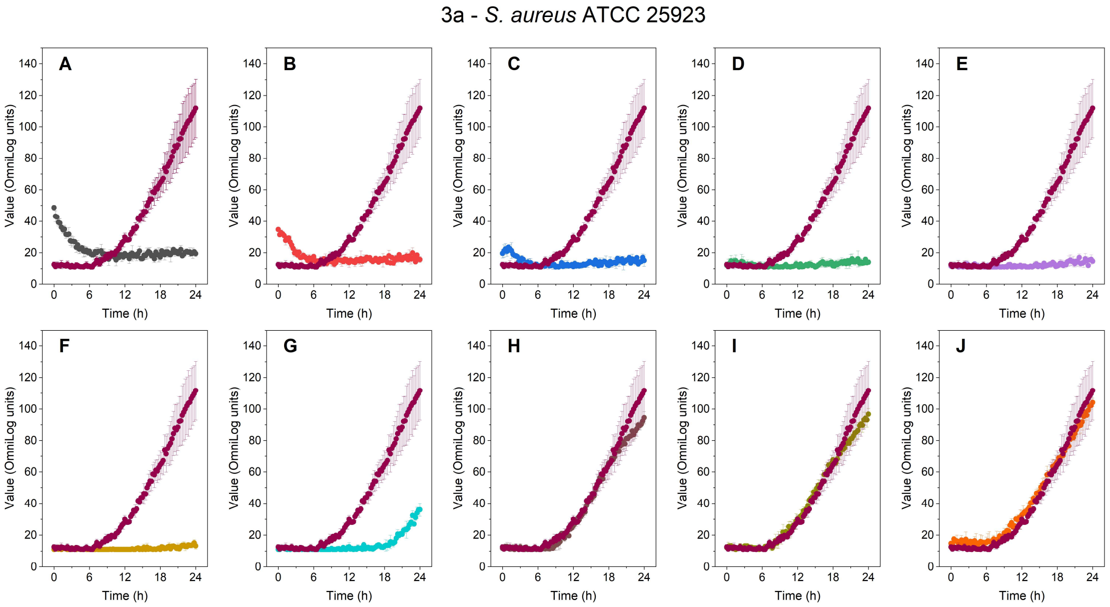

Figure S9. Determination of the MIC of **3a** against *S. aureus* ATCC 25923. A - 512 mg/L, B - 256 mg/L, C - 128 mg/L, D - 64 mg/L, E - 32 mg/L, F - 16 mg/L, G - 8 mg/L, H - 4 mg/L, I - 2 mg/L, J - 1 mg/L. The MIC was observed at 16 mg/L.

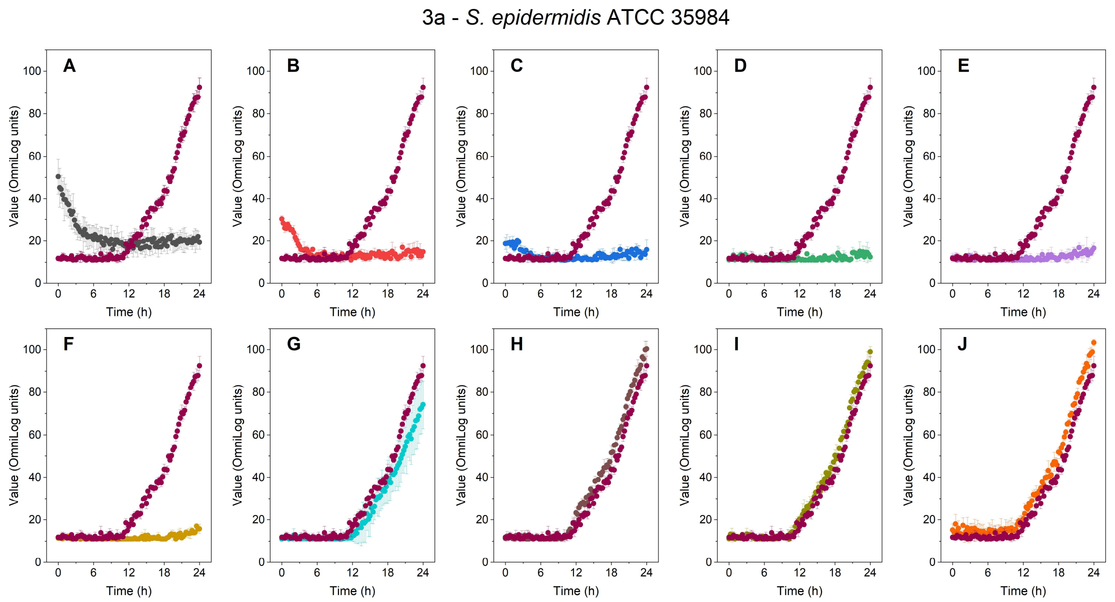

Figure S10. Determination of the MIC of **3a** against *S. epidermidis* ATCC 35984. A - 512 mg/L, B - 256 mg/L, C - 128 mg/L, D - 64 mg/L, E - 32 mg/L, F - 16 mg/L, G - 8 mg/L, H - 4 mg/L, I - 2 mg/L, J - 1 mg/L. The MIC was observed at 16 mg/L.

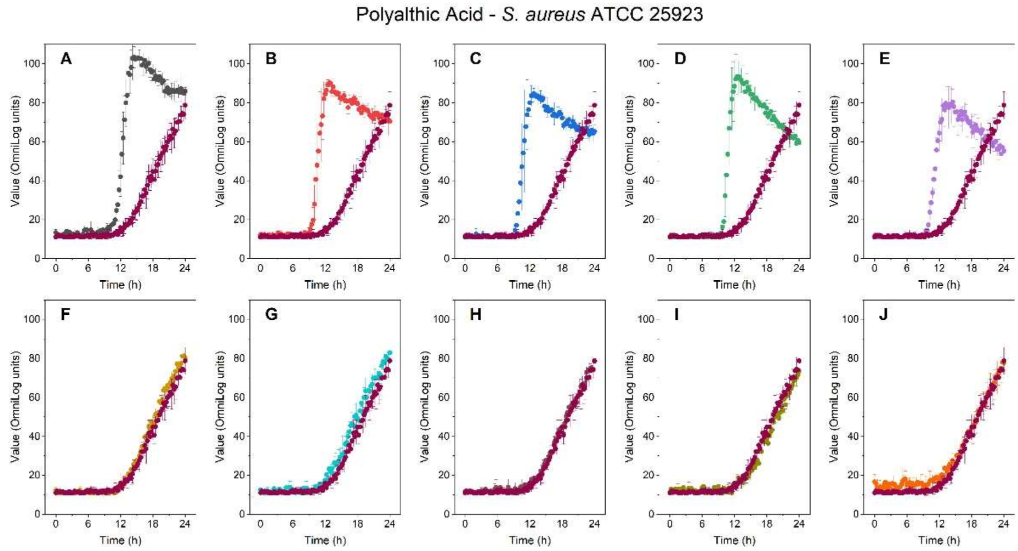

Figure S11. Determination of the minimal inhibitory concentration (MIC) of Polyalthic Acid against *S. aureus* ATCC 25923 by a kinetic growth assay. A - 512 mg/L, B - 256 mg/L, C - 128 mg/L, D - 64 mg/L, E - 32 mg/L, F - 16 mg/L, G - 8 mg/L, H - 4 mg/L, I - 2 mg/L, J - 1 mg/L. MIC was not observed (> 512 mg/L).

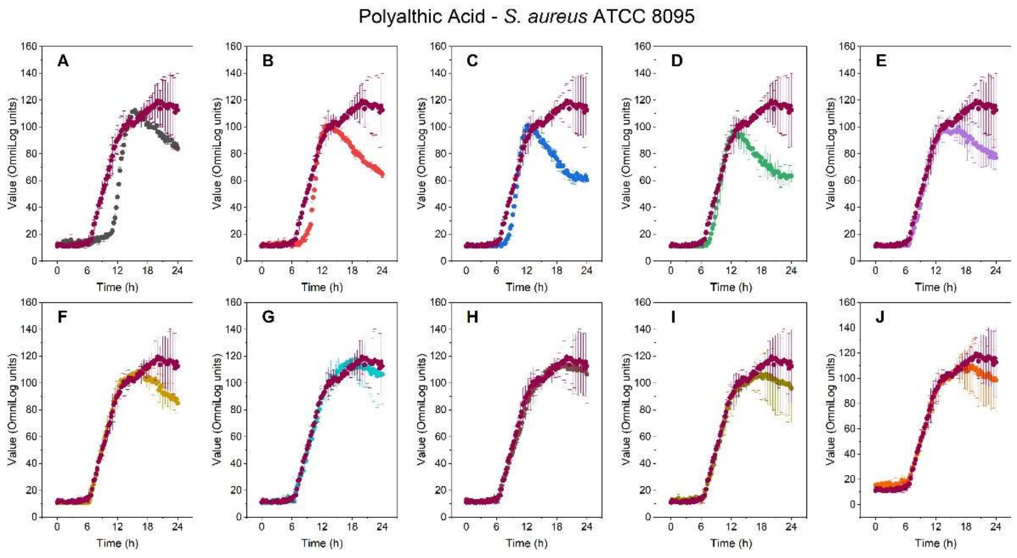

Figure S12. Determination of the minimal inhibitory concentration (MIC) of Polyalthic Acid against *S. aureus* ATCC 8095 by a kinetic growth assay. A - 512 mg/L, B - 256 mg/L, C - 128 mg/L, D - 64 mg/L, E - 32 mg/L, F - 16 mg/L, G - 8 mg/L, H - 4 mg/L, I - 2 mg/L, J - 1 mg/L. MIC was not observed (> 512 mg/L).

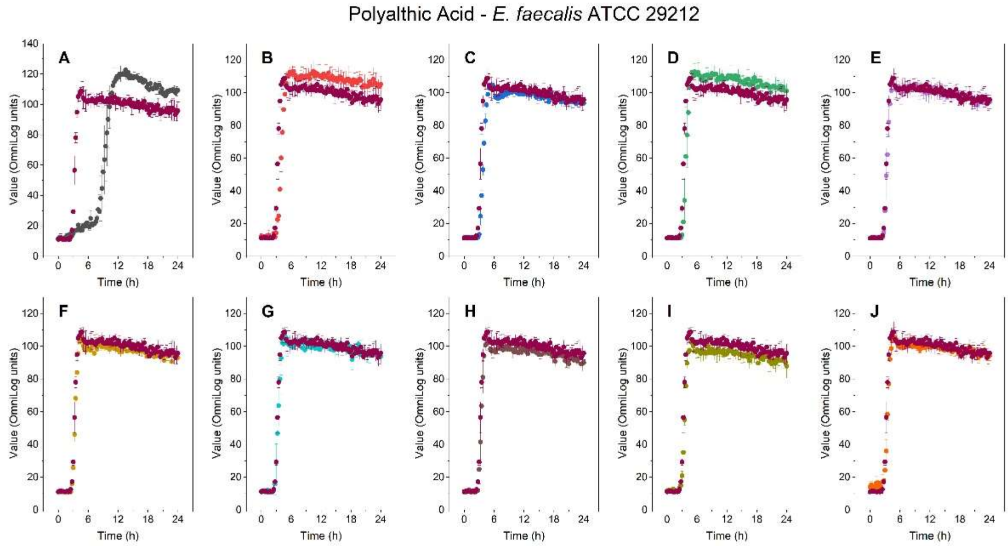

Figure S13. Determination of the minimal inhibitory concentration (MIC) of Polyalthic Acid against *E. faecalis* ATCC 29212 by a kinetic growth assay. A - 512 mg/L, B - 256 mg/L, C - 128 mg/L, D - 64 mg/L, E - 32 mg/L, F - 16 mg/L, G - 8 mg/L, H - 4 mg/L, I - 2 mg/L, J - 1 mg/L. MIC was not observed (> 512 mg/L).

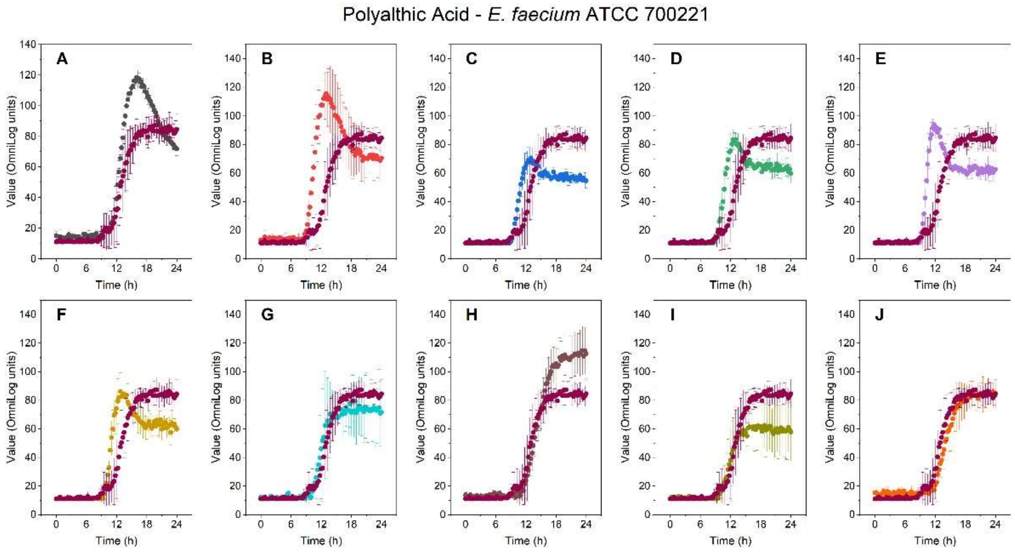

Figure S14. Determination of the minimal inhibitory concentration (MIC) of Polyalthic Acid against *E. faecium* ATCC 700221 by a kinetic growth assay. A - 512 mg/L, B - 256 mg/L, C - 128 mg/L, D - 64 mg/L, E - 32 mg/L, F - 16 mg/L, G - 8 mg/L, H - 4 mg/L, I - 2 mg/L, J - 1 mg/L. MIC was not observed (> 512 mg/L).

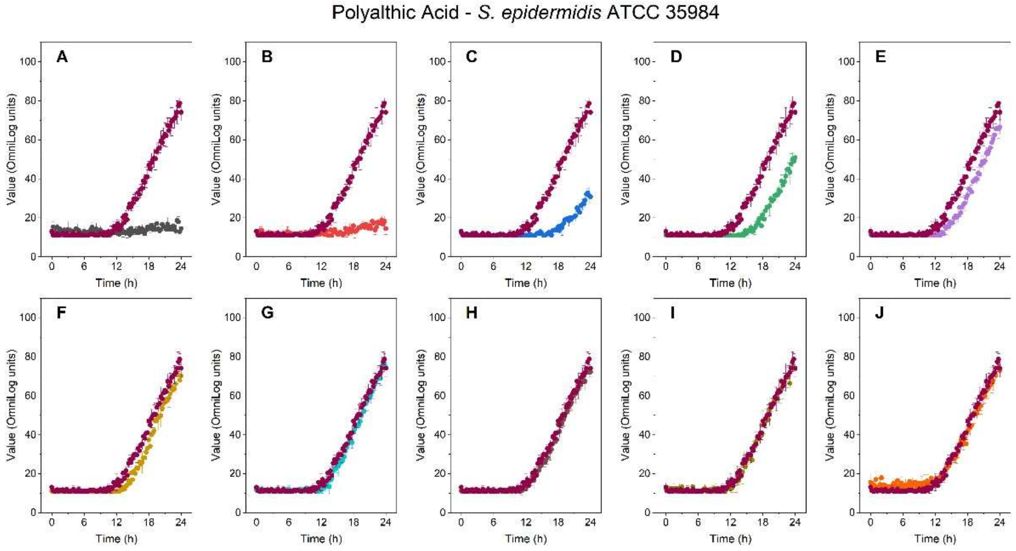

Figure S15. Determination of the minimal inhibitory concentration (MIC) of Polyalthic Acid against *S. epidermidis* ATCC 35984 by a kinetic growth assay. A - 512 mg/L, B - 256 mg/L, C - 128 mg/L, D - 64 mg/L, E - 32 mg/L, F - 16 mg/L, G - 8 mg/L, H - 4 mg/L, I - 2 mg/L, J - 1 mg/L. MIC was observed at 256 mg/L.

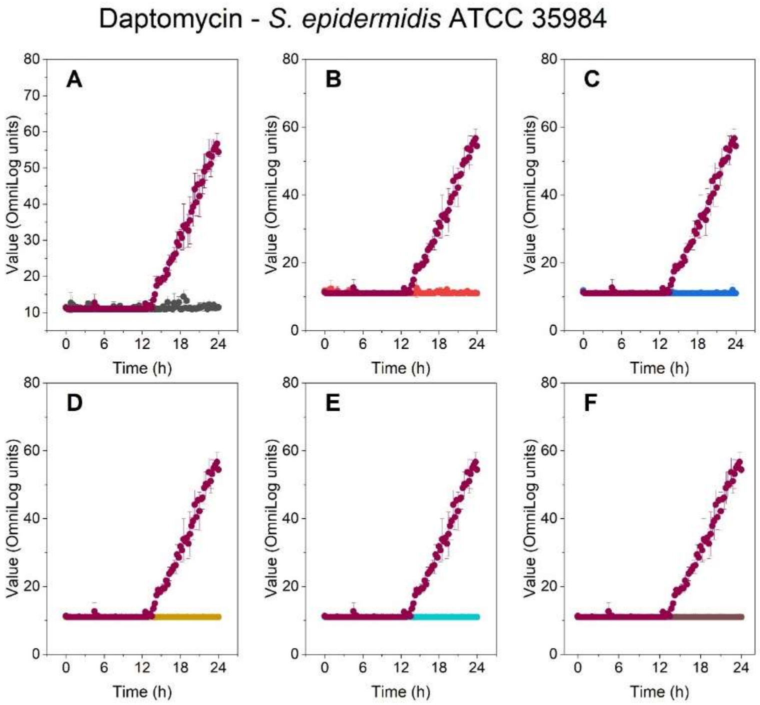

Figure S16. Determination of the minimal inhibitory concentration of daptomycin (MIC) against *S. epidermidis* ATCC 35984. A - 4 mg/L, B - 2 mg/L, C - 1 mg/L, D – 0.5 mg/L, E – 0.25 mg/L, F – 0.125 mg/L MIC was < 0.125 mg/L. Figure S11

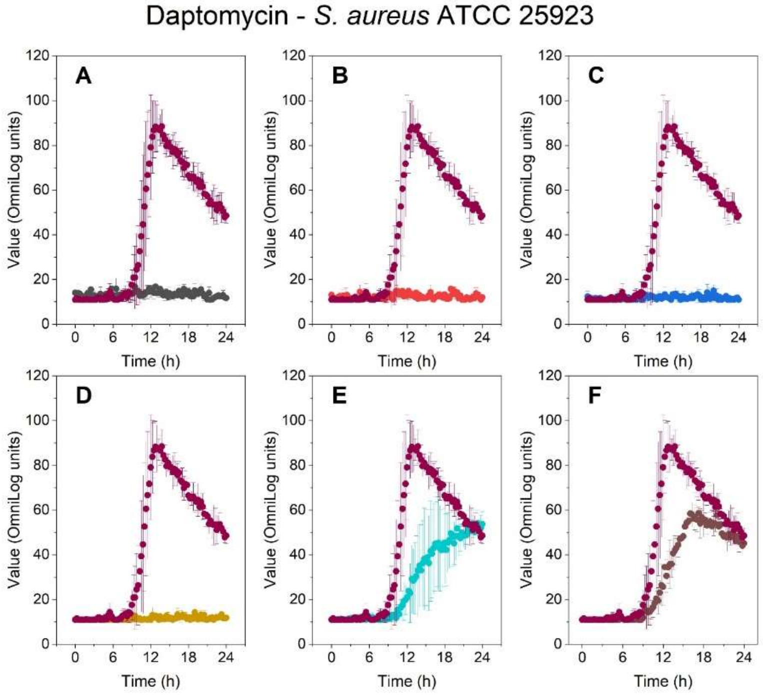

Figure S17. Determination of the minimal inhibitory concentration of daptomycin (MIC) against S. *aureus* ATCC 25923. A - 4 mg/L, B - 2 mg/L, C - 1 mg/L, D – 0.5 mg/L, E – 0.25 mg/L, F – 0.125 mg/L MIC was observed at 0.5 mg/L.

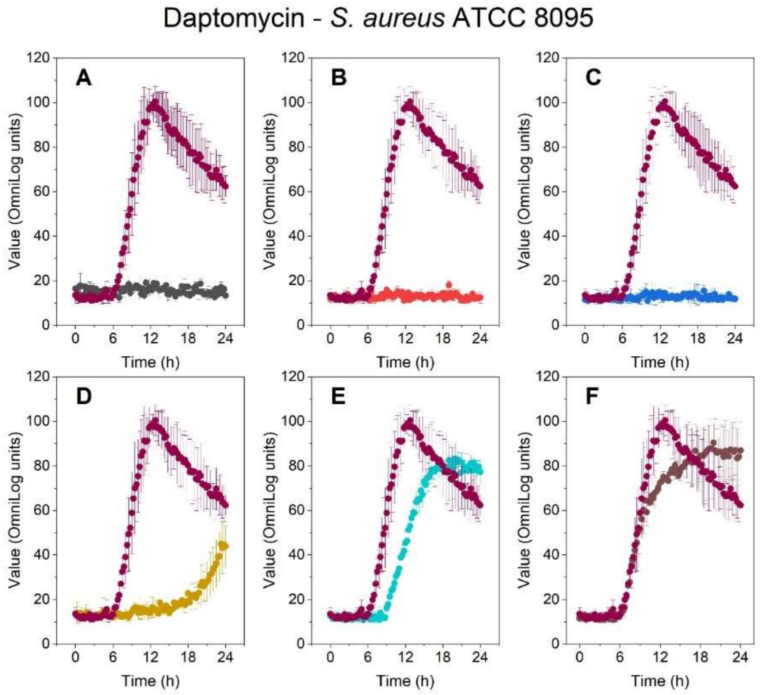

Figure S18. Determination of the minimal inhibitory concentration of daptomycin (MIC) against S. *aureus* ATCC 8095. A - 4 mg/L, B - 2 mg/L, C - 1 mg/L, D – 0.5 mg/L, E – 0.25 mg/L, F – 0.125 mg/L MIC was observed at 1 mg/L.

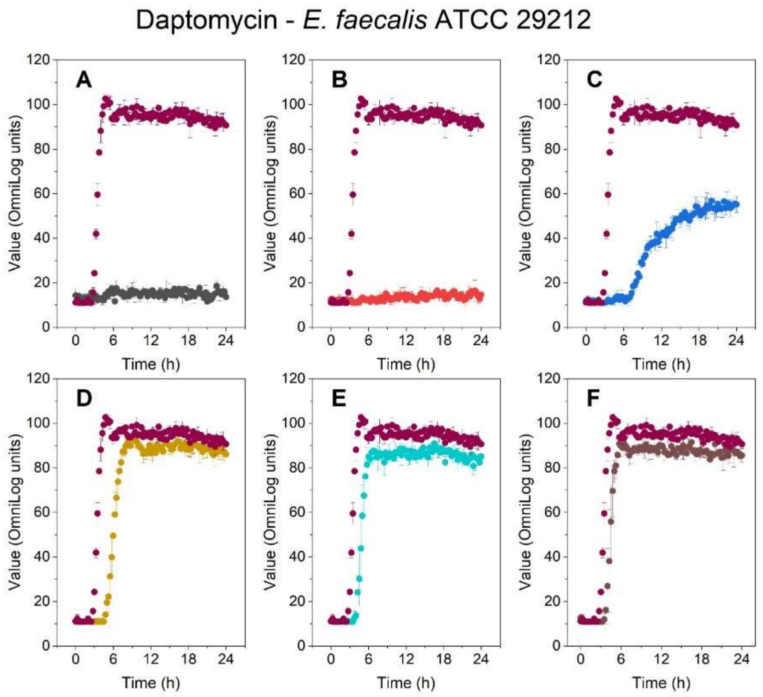

Figure S19. Determination of the minimal inhibitory concentration of daptomycin (MIC) against *E. faecalis* ATCC 29212. A - 4 mg/L, B - 2 mg/L, C - 1 mg/L, D – 0.5 mg/L, E – 0.25 mg/L, F – 0.125 mg/L MIC was observed at 2 mg/L.

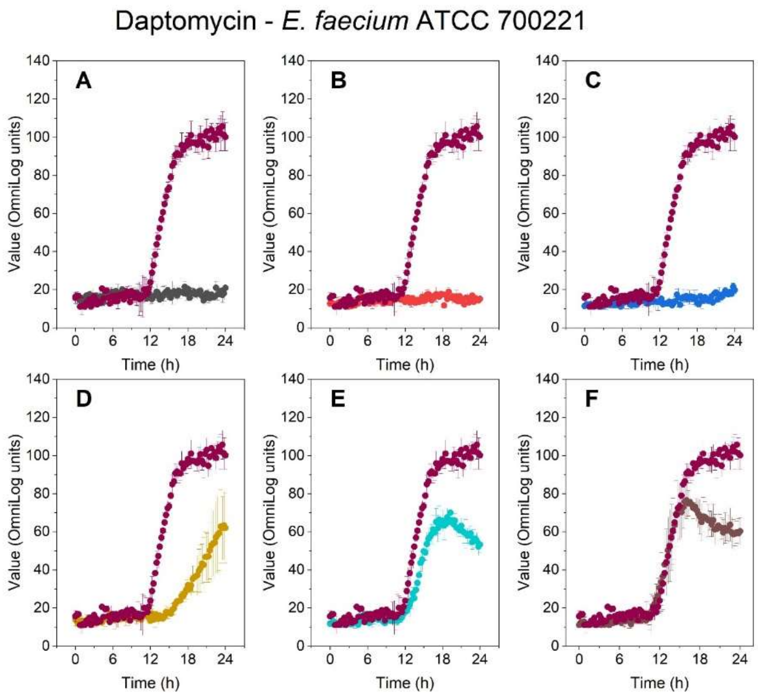

Figure S20. Determination of the minimal inhibitory concentration of daptomycin (MIC) against *E. faecium* ATCC 700221. A - 4 mg/L, B - 2 mg/L, C - 1 mg/L, D – 0.5 mg/L, E – 0.25 mg/L, F – 0.125 mg/L MIC was observed at 1 mg/L.

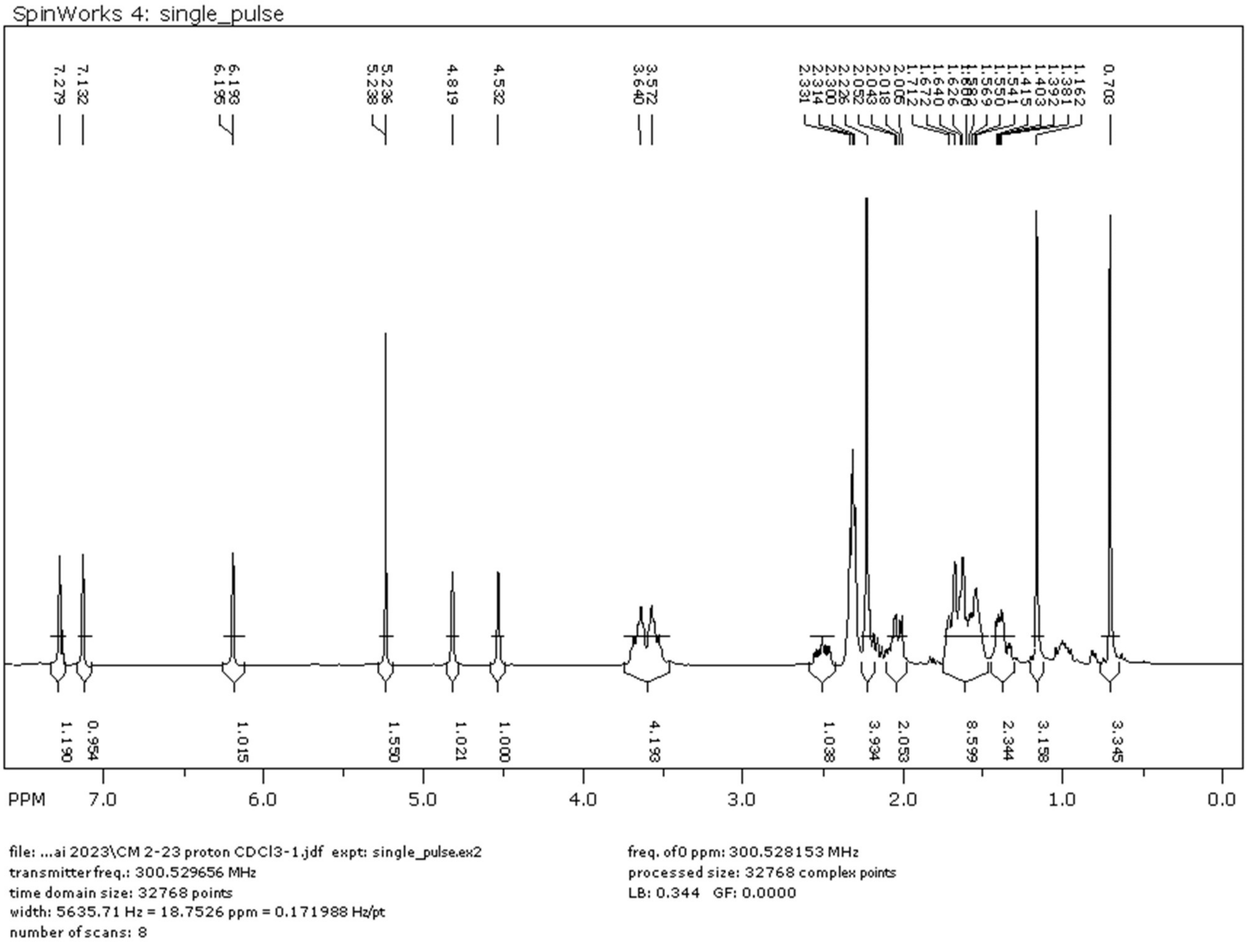

Figure S21. ^{1}H-NMR (300 MHz, $CDCl_3$) spectrum of **2a**

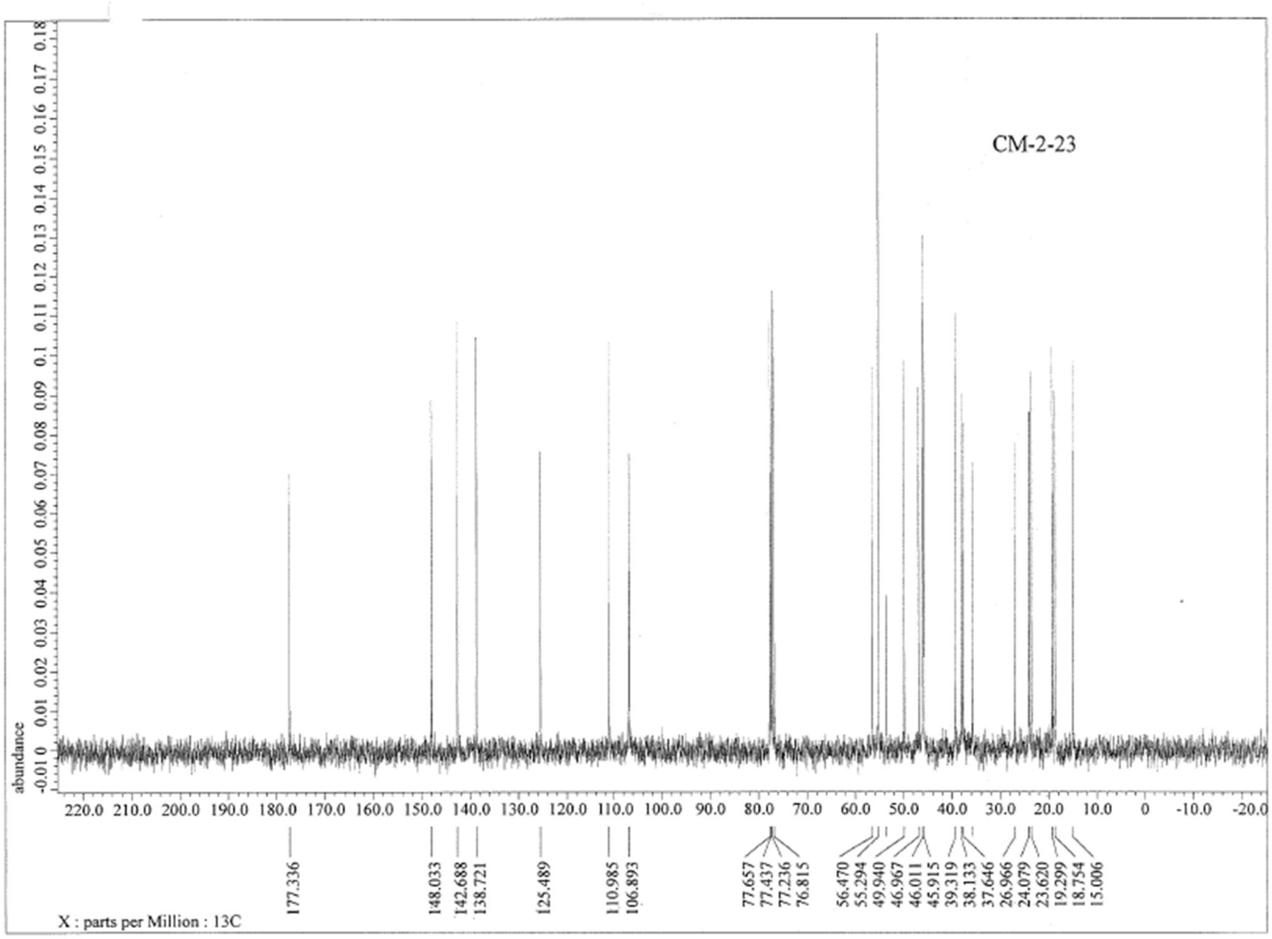

Figure S22. ^{13}C-NMR (75 MHz, $CDCl_3$) spectrum of **2a**

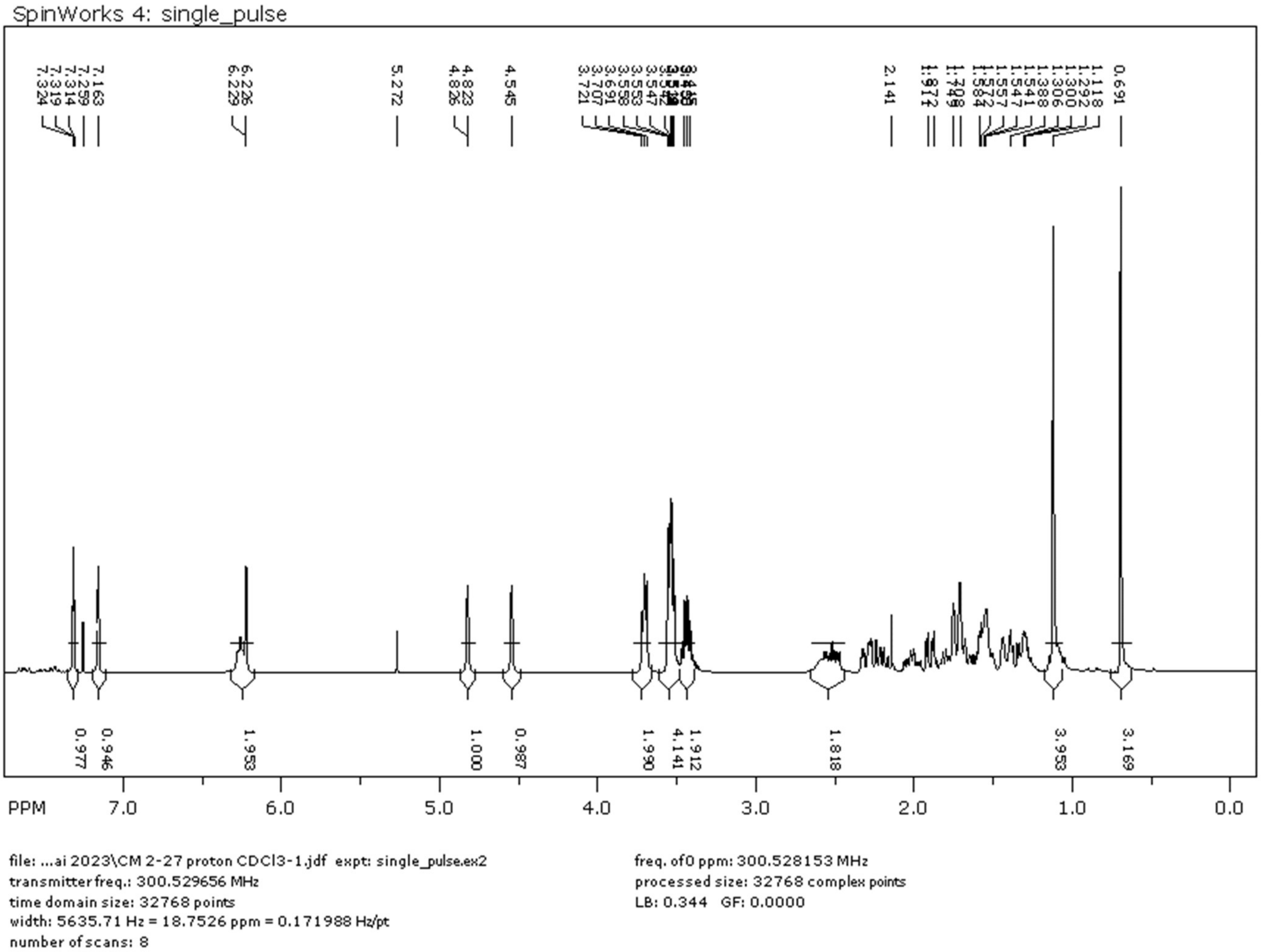

Figure S23. ^{1}H-NMR (300 MHz, $CDCl_3$) spectrum of **2b**

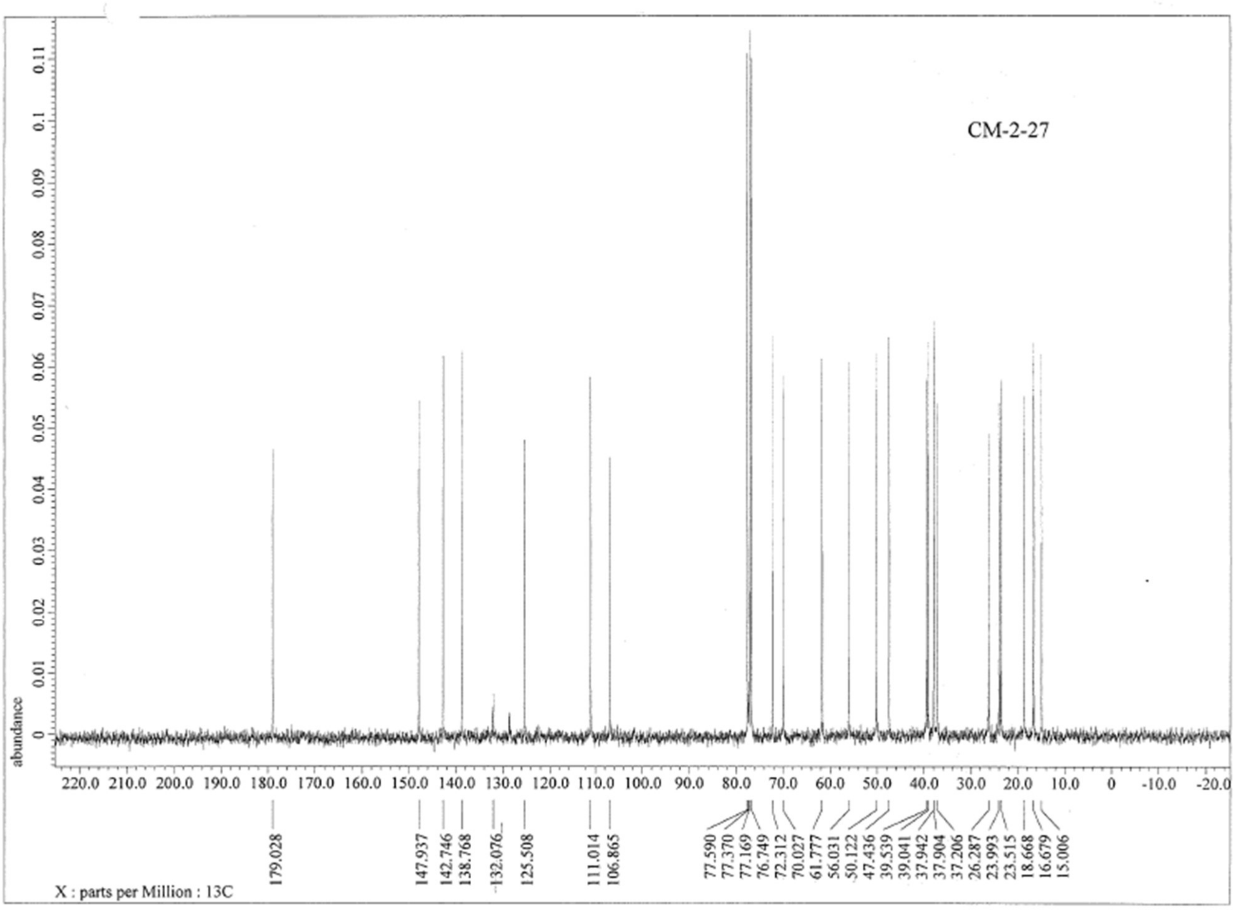

Figure S24. ^{13}C-NMR (75 MHz, $CDCl_3$) spectrum of **2b**

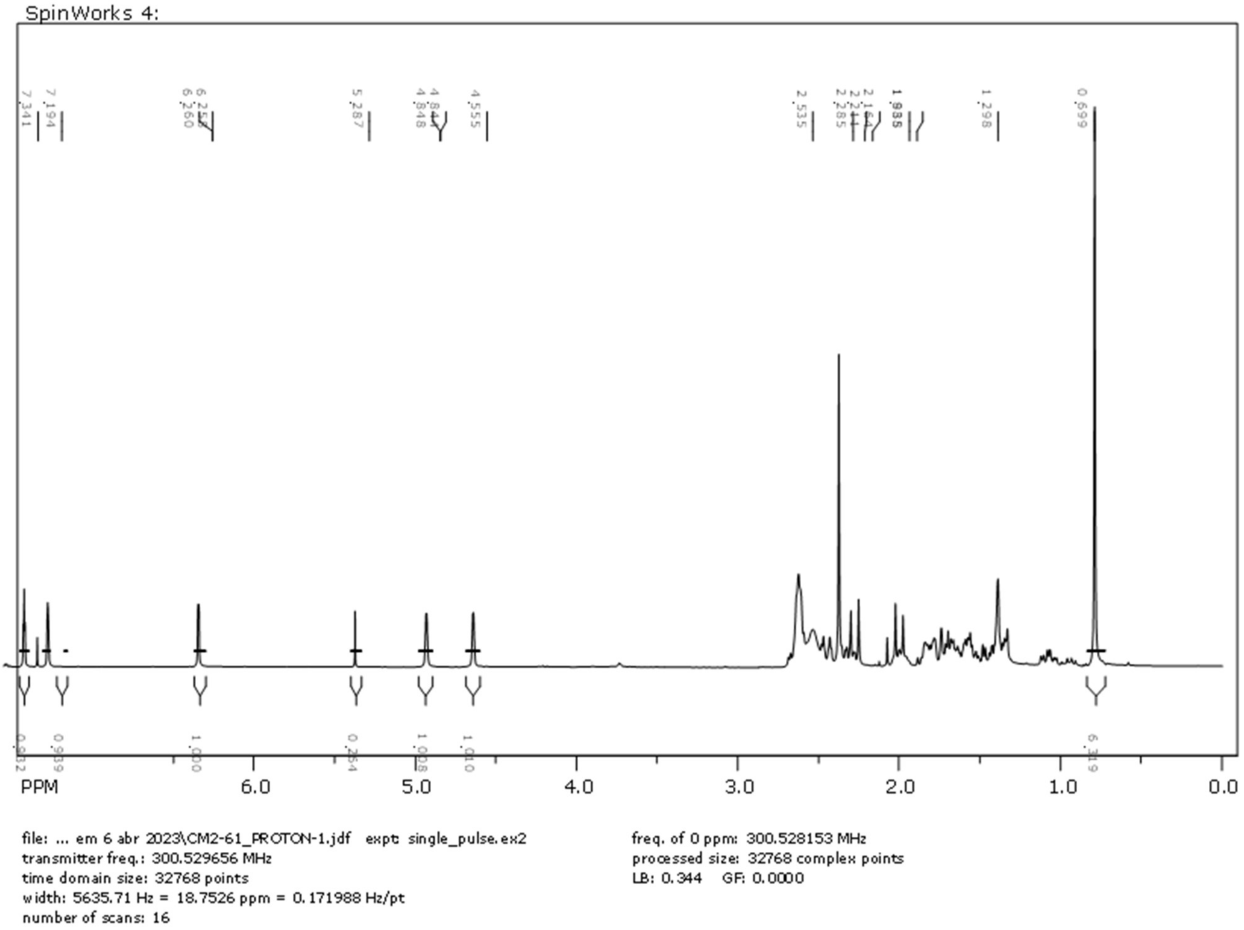

Figure S25. ^{1}H-NMR (300 MHz, $CDCl_3$) spectrum of **3a**

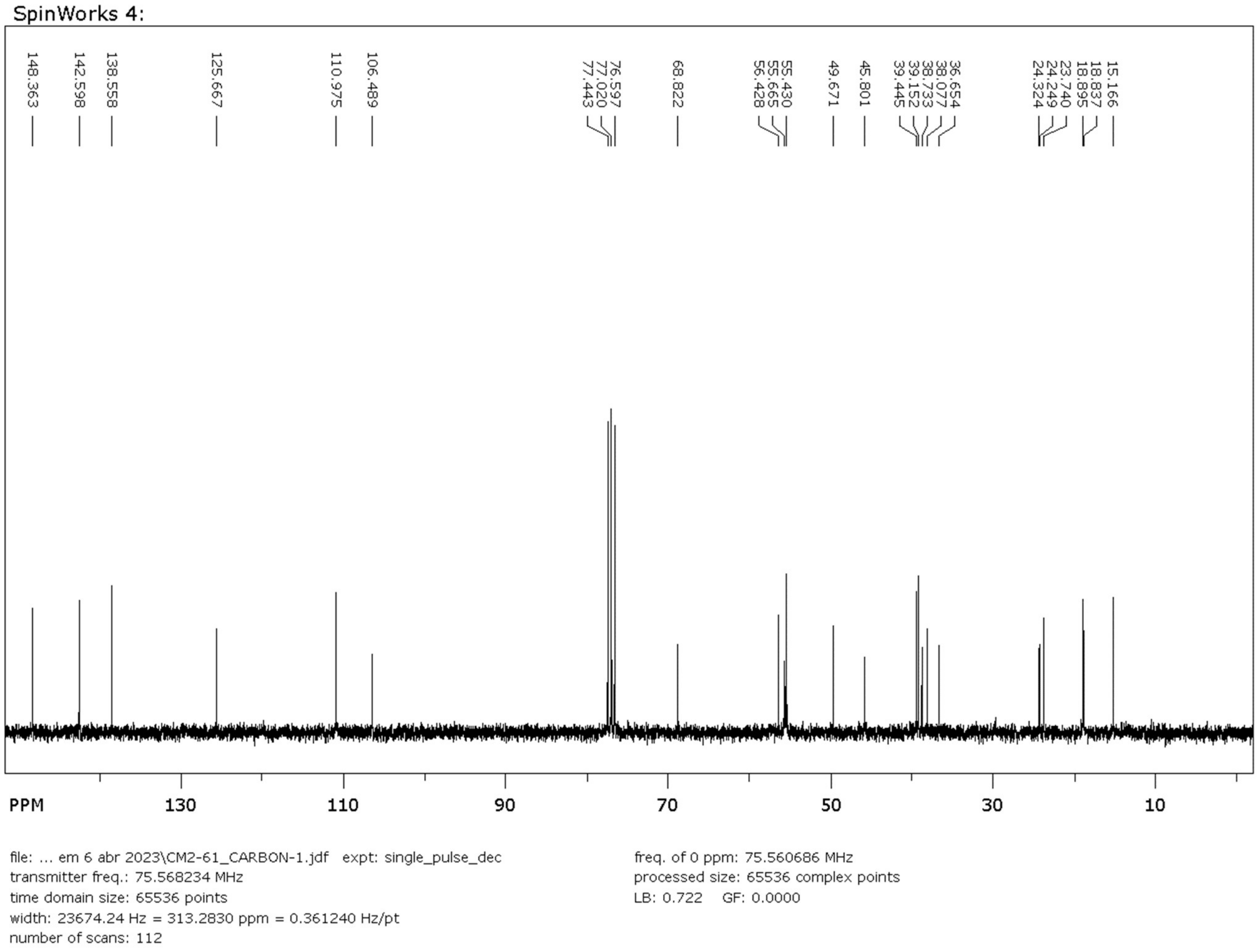

Figure S26. ^{13}C-NMR (75 MHz, $CDCl_3$) spectrum of **3a**

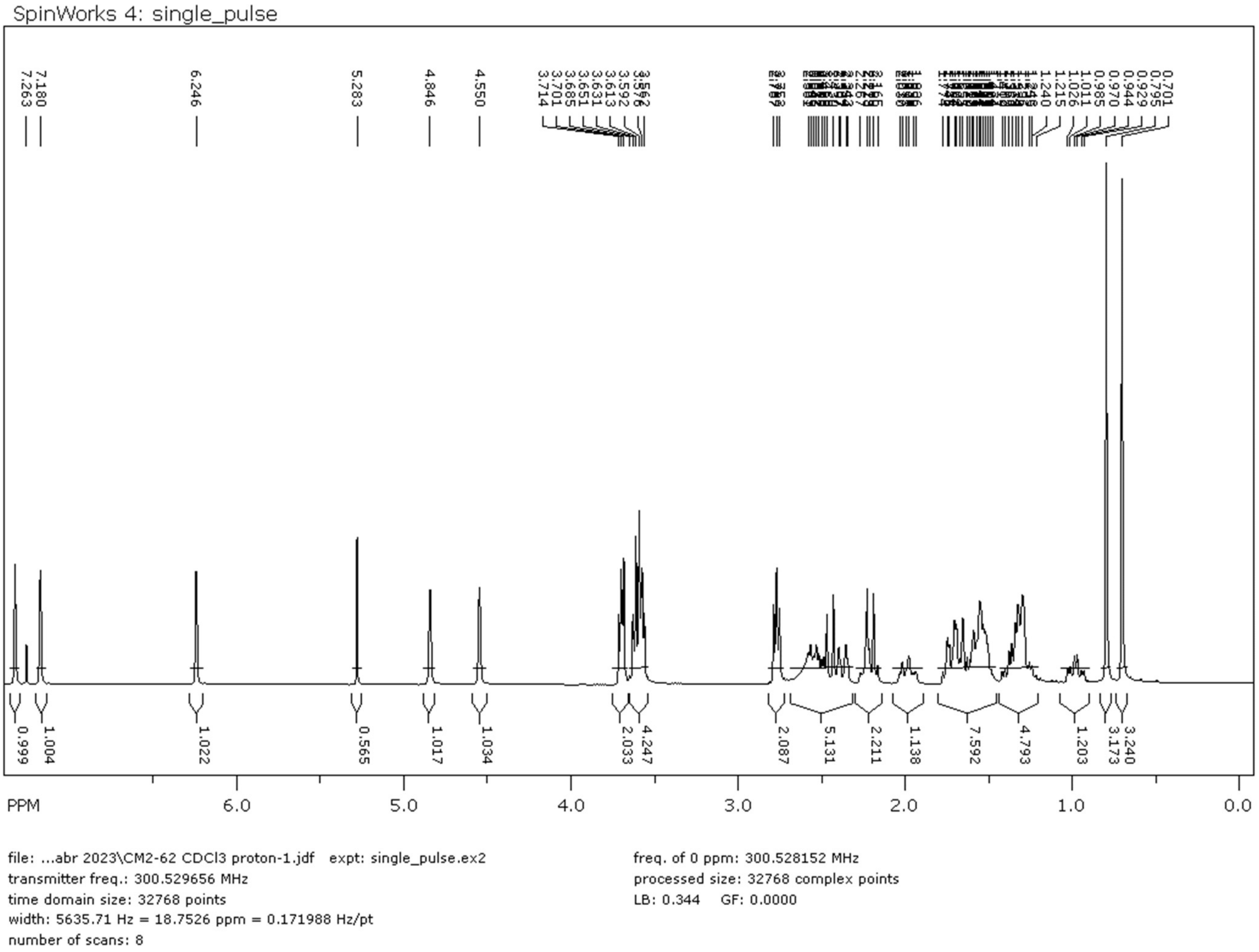

Figure S27. ^1H-NMR (300 MHz, $CDCl_3$) spectrum of **3b**

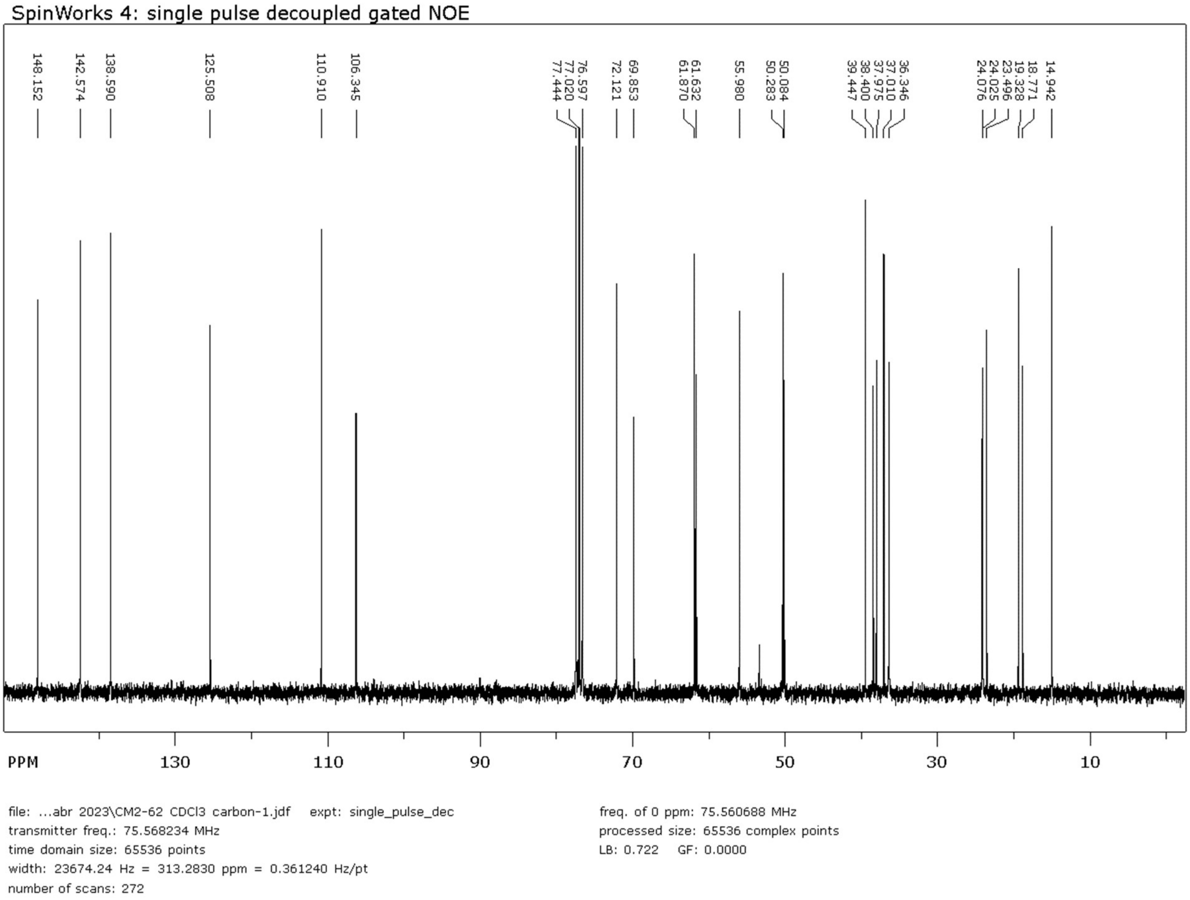

Figure S28. ^{13}C-NMR (75 MHz, $CDCl_3$) spectrum of **3b**
